# Toothbrush bristle type and toothpaste effect on surface gloss and microstructure of resin composites and zirconia

**DOI:** 10.6026/973206300213317

**Published:** 2025-09-30

**Authors:** Pooja S. Sankpal, Venkat Hemant Akurati, Manoj Kumar, Pravek Khetani, Nishtha Agrawal, Kailash L. Rathi, Heena Dixit Tiwari

**Affiliations:** 1 Department of Pedodontics and Preventive Dentistry, Bharati Vidyapeeth (Deemed to be University) Dental College and Hospital, Sangli, Maharashtra, India; 2 General Dentistry, University of Louisville, School of Dentistry, Louisville, Kentucky, USA; 3 Dental Surgeon, Health Department, State Government of Haryana, Kurukshetra, Haryana, India; 4 Department of Conservative Dentistry and Endodontics, Vyas Dental College and Hospital, Jodhpur, Rajasthan, India; 5 Department of Prosthodontics and Crown and Bridge, Government College of Dentistry, Indore, Madhya Pradesh, India; 6 Department of Orthodontics and Dentofacial Orthopaedics, S. B. Patil Dental College, Bidar, Kalaburagi, Karnataka, India; 7 Blood Cell, Commissionerate of Health and Family Welfare, Government of Telangana, Hyderabad, India

**Keywords:** Toothbrushing, resin composite, zirconia, surface gloss, *In vitro*

## Abstract

Surface gloss plays a critical role in the esthetics and longevity of restorative dental materials, yet it is often compromised by toothbrushing variables. This *in vitro* study evaluated the effect of toothbrush bristle type and toothpaste formulation on the gloss and microstructure of resin composites and zirconia fabricated through additive and subtractive techniques. Results revealed that additively manufactured zirconia exhibited the highest initial gloss and the greatest resistance to gloss loss and microstructural defects, while subtractively manufactured resin composites showed the most significant deterioration. Round-end bristles combined with whitening toothpaste produced the greatest reduction in gloss and the largest defect volumes across all materials. These findings emphasize that material selection and oral hygiene regimen are critical to maintaining the esthetic and structural integrity of restorations.

## Background:

Surface gloss of dental restorative materials contributes significantly to esthetics and durability [1]. Different toothbrush bristle geometries and toothpaste formulations may variably abrade restorative surfaces, altering gloss and microstructure [2]. Resin composites and zirconia-based materials differ in composition and manufacturing method; such differences may influence their response to mechanical and chemical challenges [3, 4]. Additively manufactured resin composites and zirconia may initially exhibit higher gloss, whereas sub-tractively manufactured counterparts may have different microstructural characteristics affecting gloss retention [5]. Toothpaste formulations with whitening or abrasive components may exacerbate gloss loss depending on material and bristle shape [6]. Simulated tooth-brushing *in vitro* allows controlled comparison of these variables and elucidation of their interactions [7, 8]. Therefore, it is of interest to evaluate the effect of toothbrush bristle type and toothpaste on surface gloss and microstructure of resin composites and zirconia.

## Materials and Methods:

Specimens (n = 160) were prepared from four material groups-additively manufactured resin composite (AM-RC), sub-tractively manufactured resin composite (SM-RC), additively manufactured zirconia (AM-Z), and sub-tractively manufactured zirconia (SM-Z). Samples were polished uniformly, and initial surface gloss was measured using a glossmeter. Each specimen was then assigned to one of four experimental subgroups combining two toothbrush bristle types (round-end vs tapered) and two toothpaste formulations (standard vs whitening). Simulated tooth-brushing was performed using 10 000 cycles in a brushing simulator under constant load. Gloss was re-measured post-brushing, and microstructural changes were assessed via µ-CT at high resolution. Data were analyzed using three-way ANOVA and one-way ANOVA, with significance set at α = 0.05.

## Results:

Post-brushing gloss values (mean ± SD) are presented in Table 1 (see PDF). Initial gloss values ranged from 85.3 ± 4.1 GU for AM-Z to 68.7 ± 3.9 GU for SM-RC. After brushing, AM-Z retained gloss at 74.2 ± 5.0 GU (a decline of ~12.9 GU), while SM-RC dropped to 45.1 ± 4.8 GU (~23.6 GU loss). Round-end bristles with whitening toothpaste produced the greatest gloss loss across all materials. Micro-CT analysis (Table 2 - see PDF) showed minimal subsurface alterations in AM-Z (mean defect volume 0.15 mm^3^), whereas SM-RC exhibited larger microstructural defects (0.45 mm^3^). Whitening toothpaste combined with round-end bristles induced the largest defect volumes. Three-way ANOVA revealed that material type (p < 0.001), bristle type (p = 0.02), and toothpaste formulation (p = 0.01) all had significant effects on gloss retention. One-way ANOVA confirmed that round-end bristles and whitening toothpaste caused significantly greater gloss loss than other combinations (p < 0.05).

## Discussion:

The present *in vitro* study demonstrates that material type, toothbrush bristle geometry, and toothpaste formulation substantially affect surface gloss retention and microstructure of resin composites and zirconia. Additively manufactured zirconia (AM-Z) exhibited superior initial gloss and better retention following brushing, aligning with recent findings showing that specific fabrication and polishing protocols yield higher gloss in zirconia [9, 10]. Ultra-translucent zirconia polished with diamond rubber abrasives maintained or even improved surface gloss under brushing, whereas glazing protocols showed greater degradation [9]. This supports our observation that AM-Z sustains gloss more effectively under mechanical challenge. Resin composites generally showed greater gloss loss and microstructural defects, consistent with reports that composite materials are susceptible to surface degradation during tooth-brushing simulation, particularly with abrasive toothpaste [11, 12]. Studies comparing nan-ohybrid and bulk-fill composites brushed with whitening dentifrices found variable outcomes in gloss and hardness, emphasizing the role of formulation and filler characteristics [11]. The significant gloss decline in SM-RC aligns with such variable resistance among resin composites. Toothbrushing parameters critically influence material response. Our use of round-end bristles with whitening toothpaste yielded markedly higher gloss loss, confirming that bristle tip geometry and toothpaste abrasiveness synergistically exacerbate surface wear. Prior evidence suggests whitening formulations and abrasive brush designs can lead to more pronounced surface changes [13], reinforcing the need for careful toothbrush-toothpaste pairing. Micro-CT allowed quantification of subsurface microstructural changes, which are less well explored in comparative studies. The larger defect volumes in SM-RC indicate that some manufacturing and material combinations are more prone to mechanical-chemical disruption beneath the surface. While many studies focus on surface roughness and gloss, our findings highlight subsurface microstructural integrity as a critical consideration in restorative longevity [13, 14-15]. Clinically, these results suggest that material selection (favoring AM-Z) and patient guidance on toothbrush and toothpaste choice may mitigate surface degradation of restorations. The superior performance of AM-Z might reflect its homogeneous microstructure, higher filler density, and optimized surface finish; further work should explore its resilience under longer brushing cycles and different abrasives. Limitations of this study include it *in vitro* design and controlled brushing regimen that May not fully replicate *in vivo* variability in force, brushing angle, and slurry dynamics. Moreover, micro-CT sensitivity may vary with material radiopacity, potentially influencing defect detection. Future research should extend to longer brushing durations, different composite formulations, and clinical correlation of gloss loss with patient perception. In summary, our data show that additively manufactured zirconia offers greater resistance to gloss reduction and microstructural damage during brushing. Material-specific susceptibility, especially in resin composites, underscores the importance of selecting appropriate materials and oral hygiene tools to preserve restoration esthetics and integrity.

## Conclusion:

Additively manufactured zirconia demonstrates superior surface gloss retention and minimal microstructural damage under simulated tooth-brushing. Toothbrush bristle shape and toothpaste formulation importantly influence wear, with round-end bristles and whitening toothpaste causing greatest degradation. Choice of material and hygiene regimen should be considered to optimize restorative longevity.
